# Tuning the electrical properties of the p-type transparent conducting oxide Cu_1−x_Cr_1+x_O_2_ by controlled annealing

**DOI:** 10.1038/s41598-018-25659-3

**Published:** 2018-05-08

**Authors:** P. Lunca-Popa, J. Afonso, P. Grysan, J. Crêpellière, R. Leturcq, D. Lenoble

**Affiliations:** Materials Research and Technology Department (MRT), Luxembourg Institute of Science and Technology (LIST), 41 rue de Brill, L-4422 Belvaux, Luxembourg

## Abstract

Off-stoichiometric copper chromium oxide delafossite received lately a great interest due to its high p-type electrical conductivity and adequate optical transmittance in the visible range. However, for a suitable integration in active devices such as p-n junctions, transistors or optoelectronic devices, the electronic properties must be efficiently tailored. Here, post-deposition thermal treatment is proven as an adequate approach for finely controlling the electrical properties of this former degenerate semiconducting material. The energetics of the annealing process are investigated using two different approaches, as a function of the annealing temperature and as a function of the annealing time, allowing the accurate determination of the activation energy of the annealing of defects. By using this method, the electrical carrier concentration was varied in the 10^21^ – 10^17^ cm^−3^ range while the recorded changes in the drift mobility covered three orders of magnitude. Moreover, we demonstrate the ability to accurately manipulate the Fermi level of such materials, which is of great importance in controlling the carrier injection and extraction in optoelectronic active layers.

## Introduction

In the field of transparent conductive oxides (TCOs) copper-based delafossite materials are promising candidates as p-type transparent semiconductors closing the gap of optical and electrical properties towards current standard n-type semiconductors (a transmittance greater than 80% in the visible range and an electric conductivity up to 1000 S cm^−1^). The interest in these peculiar delafossite compounds was ignited after the report of CuAlO_2_ as a first p-type semiconductor with decent optical transparency^1^ in the visible range and was reinforced after reporting a breakthrough of electrical conductivity up to 220 S cm^−1^ obtained for Mg-doped CuCrO_2_^2^. Various copper-based delafossites CuMO_2_ (M = Cu, Cr, Ga, In, Fe, B) were thoroughly studied^3^ in the effort to understand the rationales of the p-type conductivity and the electrical transport mechanism within for optimizing subsequently theirs electrical and optical properties. Copper vacancies^4^ or oxygen interstitials^1,5^ were mainly suggested as the p-type doping source whilst small polaron^6,7^ or band conduction models^8,9^ were proposed to elucidate the conduction mechanism in such materials. Moreover, recent reports had shown large conductivity (larger than 10 S cm^−1^) and adequate transparency for highly off-stoichiometric copper chromium delafossites.^10–13^ In these particular compounds the crystalline phase of delafossite is preserved although a copper deficiency up to 33% is observed.

In our previous work^14^ we reported the synthesis and the characterization of such off-stoichiometric Cu-Cr-O delafossite thin films with conductivities greater than 100 S cm^−1^ and optical transmittances up to 50%. Finite lines of copper vacancies chains randomly distributed within crystalline grains were observed (in Transmission Electron Microscopy) and furthermore suggested as the possible source of high doping in as-deposited films. A peculiar stoichiometry with a 33% copper deficiency compensated by a surplus of 33% chromium (i.e. Cu_0_._66_Cr_1.33_O_2_) was found in both as-deposited and annealed (900 °C) films whilst the delafossite crystalline structure remains unaltered. However, after annealing a drop of the carrier’s concentration from 10^21^ to 10^17^ cm^−3^ or even lower was measured and associated with the healing of the chained defects. We then suggested that the main driving force leading the changes in the defect chemistry is the “healing” of defects, a process driven by short-range structural changes.

In the present paper we further investigate how a controlled post-deposition thermal treatment can be used as a very effective approach for tailoring electrical properties of Cu_0.66_Cr_1.33_O_2_. Such fine tuning is required for the electrical engineering of transparent devices such as p-n junctions or p-type transistors^15^. In order to achieve this goal, the metastable nature of the Cu-vacancies chains described above was investigated. Two different types of thermal treatments were used in the present study in order to investigate the thermodynamics of the annealing process: different annealing times at a fixed temperature (900 °C) or a fixed amount of time (900 s in this case) at various temperatures (from 650 °C to 850 °C). The first treatment demonstrates conditions for a fast process which is more adequate to technological applications where long lasting processes might be considered costly. The temperature is situated safely lower than 1100 °C, the reported limit of the materials stability for the copper delafossite phase^16^. The second treatment, involving lower temperatures, allows a better control due to the smooth variation of electrical properties. The experimental results show that the controlled thermal treatment can be used as a versatile process for controlling the holes concentration, the electrical mobility or even the electronic work function, all being key features for the engineering of electronic properties of transparent solid state devices.

## Results and Discussions

**Figure 1:**
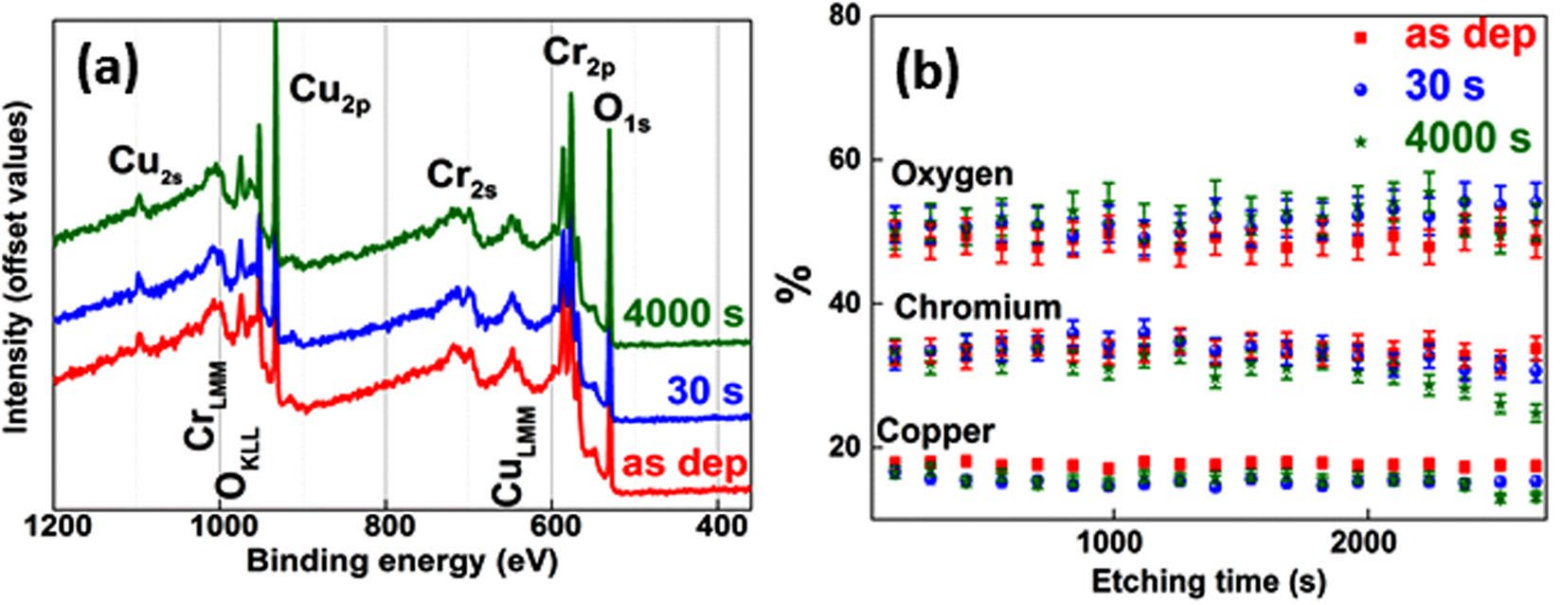
**(a)** Full XPS spectra for as-deposited and annealed films for 30 and respectively 4000 s; **(b)** measured chemical composition for same films; Annealing temperature: 900 °C.

We start by investigating the chemical composition of as-deposited and annealed films for various annealing time intervals in order to confirm the stability of the material’s chemistry upon thermal treatment. Figure 1(a) depicts XPS results for as-deposited films and for films annealed for 30 s and respectively 4000 s. The XPS spectra look similar, suggesting no major changes of chemical concentrations. Besides XPS characteristic peaks for Cu_(2p,2s)_, Cr_(2p,2s)_ and O_1s_, Auger O_KLL_ Cu_LMM_ and Cr_LMM_ peaks are present in the spectra^17^. The positions of Cu_2p_ peaks (1/2 at 932.6 eV and 3/2 at 952.5 eV) do not vary upon annealing. The distance between them is 19.9 eV, a clear indication of the delafossite phase. No satellite peak of Cu is observed, confirming that only Cu in +1 oxidation state is detected by XPS^13^. The Cr_2p_ peaks are observed at binding energies of 576.6 eV (3/2) and 585.6 eV (1/2) respectively. The distance between Cr_2p_ and O_1s_ remains at a constant value of 45.3 eV for all samples. Moreover, the Auger Cu_LMM_ peak observed at a binding energy of 568.6 eV confirms the purity of our delafossite phase^18^.

The chemical compositions for as-deposited and annealed films are shown in Fig. 1(b). Concentrations around 16, 33 and 50% are measured (within the XPS measurement accuracy) for Cu, Cr and O respectively. This peculiar stoichiometry may lead to deviations in the partial charge of each atom as compared to the stoichiometric CuCrO_2._ The bounds are known to be partially ionic and partially covalent and consequently the oxidation state of each atom in the crystal is not directly equal to the most stable oxidation state of each atom^19^. The X-Ray Diffraction, XRD, (Figure S1) analysis confirms furthermore the preservation of the delafossite phase after thermal treatments along with an increase of the crystalline grains’ size and a relaxation of c lattice parameter (Figure S2). The change of the grains’ size is also visible in AFM scans presented in Figure S3 from the supplementary information section.

Six samples with similar (averaged value: 15.6 ± 6.9 S cm^−1^) conductivities (Table 1) were used for each thermal treatment study (one was kept as reference in each case). Electrical conductivity values of tens of S cm^−1^ are obtained using the set of deposition’s parameters described within the experimental methods section. This level of conductivities is usually reported for off-stoichiometric delafossites thin- films fabricated using non-equilibrium chemical methods as dynamic liquid injection chemical vapour deposition^10,11,14^ or chemical spray deposition^12,13^. These values are significantly higher than those reported for intrinsic^20,21^ or some doped^22,23^ CuCrO_2_ delafossites thin-films.

**Table 1:** Annealing times or annealing temperatures, initial electrical conductivities and the ratios after thermal treatment, Seebeck coefficients and calculated carrier’s concentration and mobility (within small polaron model) for: left - films annealed for 900 s at different temperatures (*sample annealed for 3600 s); right - films annealed at 900 °C for different time intervals. NA – measures beyond the sensitivity of our apparatus.

t °C	σ_0_ S/cm	σ_0_ σ_f_	S µVK^−1^	p cm^−3^	µ cm^2^V^−1^s^−1^	t (s)	σ_0_ S/cm	σ_0_ σ_f_	S µVK^−1^	p cm^−3^	µ cm^2^V^−1^s^−1^
	15	1	100	1.7E + 21	0.08	0	17	1	110	1.5E + 21	0.070
650*	12	3	128	1.2E + 21	0.004	30	10	128	340	1.1E + 20	0.002
700	11	102	184	6.6E + 20	0.001	60	10	173	360	8.9E + 19	0.004
750	31	5500	257	2.9E + 20	0.002	200	7	4700	850	3.0E + 17	0.031
800	21	14000	374	7.3E + 19	0.0001	1000	19	12600	947	9.4E + 16	0.086
850	23	54000	753	9.3E + 17	0.007	4000	11	NA	NA	NA	NA

**Figure 2:**
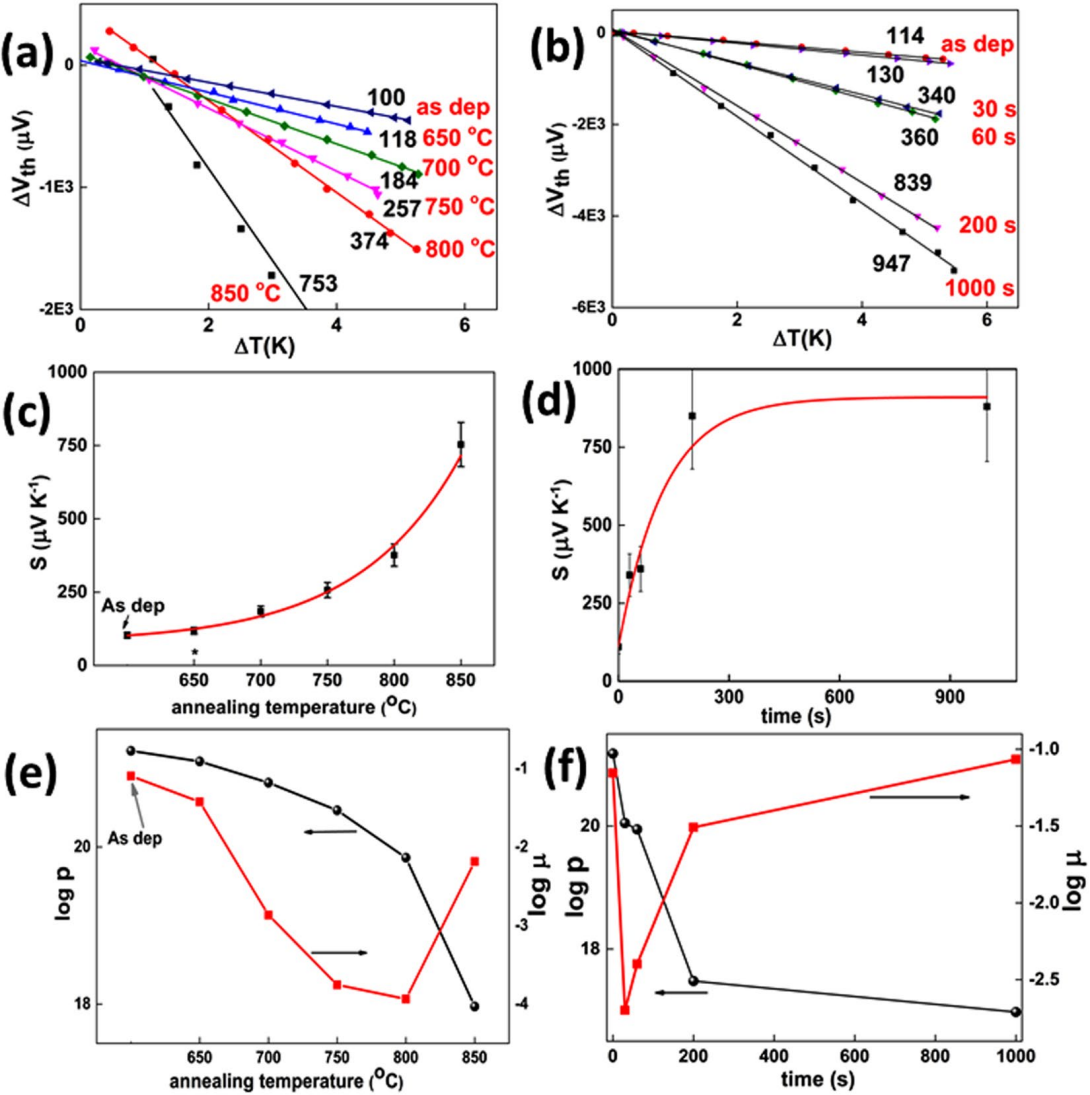
Seebeck coefficient measurement for Cu-Cr-O thin films **(a)** annealed at different temperatures; **(b)** annealed at 900 °C for various time intervals. Thermoelectric voltage ΔV_th_ = V_hot_ − V_cold_ is measured as a function of the temperature gradient ΔT applied across the sample, with copper wire as reference. The measurement has been performed with the cold contact maintained at room temperature (23 °C). The values of the Seebeck coefficient S = −ΔV/ΔT, determined from a linear fit, are indicated on the graph for each sample. The voltage offset is due to parasitic offsets from the measurement apparatus due to samples’ high resistance; **(c)** Seebeck coefficient vs annealing temperature; **(d)** Seebeck coefficient vs annealing time for thermal treatments at 900 °C. Red curves are corresponding to exponential fits; Logarithm of the carrier concentration (black) and of drift mobility (red) **(e)** as function of annealing time for samples annealed at a fixed temperature 900 °C; **(f)** as function of the annealing temperature for samples annealed for a fixed time t = 900 s.

Five of the samples were heated for 900 s at 650, 700, 750, 800 and 850 °C, respectively. For the first sample heated at 650 °C no change was observed after 900 s and consequently the time was furthermore increased up to 3600 s when a three times diminution of the electrical conductivity (**σ**_**0**_**/ σ**_**f**_**)** was finally observed. This is in agreement with the work of Gotzendorfer *et al*.^23^ where changes in electrical properties of CuCrO_2_ were observed starting from temperatures around 620 °C. Second set of samples were heated at 900 °C for 30, 60, 200, 1000 and 4000 s respectively. For the last sample the measured conductivity was beyond the sensitivity of our apparatus (10^−4^ S cm^−1^). For each sample the Seebeck coefficient and the conductivity were measured before and after the thermal treatment and the results are presented in Fig. 2 and Table 1. The first observation is the negative slope of ∆V_th_/∆T further proving the p-type nature of our films. For as-deposited samples values of S around 100–130 µV K^−1^ were measured. For the sample heated at 650 °C a similar value (118 µV K^−1^) of the Seebeck coefficient (Fig. 2(a)) is measured evidencing a slow healing of Cu-vacancies chains defects at this temperature. Beyond 700 °C important changes appear after 900 s of annealing. The electrical conductivity decreases monotonously with the annealing temperature until a diminution of 50 000 times, as measured in the case of the sample heated at 850 °C. The Seebeck coefficient follows an exponential decay (Fig. 2(c)) with the annealing temperature. This coefficient is directly related to the carriers’ concentration which is further dependent on the concentration of defects. In the material investigated here, we have previously demonstrated that the defects are the chains of Cu vacancies observed by TEM experiments^14^. Therefore we may correlate the decrease of the carriers’ concentration with the annihilation of these chains of Cu vacancies. A further indication will be presented later when analysing the thermodynamics of the annihilation reaction. The exponential decay confirms the metastable nature of defects and that a thermal activated law governs their healing. Similar healing of defects is observed when using the fast annealing process. The conductivity decreases by two orders of magnitude during short (30–60 s) thermal treatments, followed by a continuous decrease with the annealing time down to the 10^−4^ S cm^−1^ range for the sample heated during 4000 s. The values of the Seebeck coefficient can be regrouped in three main regimes as depicted in Fig. 2(b). Values around 100–130 µV^−1^ are measured for as-deposited high conductive samples. Similar values are usually reported for off-stoichiometric or doped delafossite thin-films with electrical conductivities around 10 S cm^−1^ or higher^2,10–14,24^. After short annealing times (up to 60 s) the coefficient increases up to values around 360 µV K^−1^. Further increase of the annealing time results in values for Seebeck coefficient of 850 µV K^−1^, similar with values reported for non-doped CuCrO_2_^25^. Again the dependence of the Seebeck coefficient on the annealing time is exponential (Fig. 2(d)). It was previously reported^26,27^ that performing Hall measurements on copper p-type delafossites is challenging due to very low values of mobility. Furthermore collected Hall voltage values are comparable with the background signal. Hall measurements at high magnetic fields (up to 9 T) were performed on our as-deposited sample and resulted in highly scattered data points^28^. An averaged value of 0.01 cm^2^ V^−1^s^−1^ can be extracted after making a large set of assumptions. This averaged value matches the mobility value as we extract it from our Seebeck measurements. This makes us confident that the combined Seebeck and conductivity measurements provide reliable values of electronic properties of our materials. From the electrical conductivity and the Seebeck coefficient measurements we extract the carriers’ concentration of thin films. It is important to distinguish on one side the origin of holes in our system, which are related to peculiar defects (here chains of Cu vacancies), and on the other side the localisation of the free (mobile) holes in our system, which is related to the orbitals constituting the valence band maximum. In Cu-based delafossite materials, it has been widely assumed that the valence band is mainly made from d orbitals of Cu atoms, leading to a transition of the oxidation state of Cu^I^ to Cu^II^. In conclusion whatever the origin of the holes (chains of Cu vacancies, isolated Cu vacancies, O interstitials, extrinsic dopants etc.), the free holes concentration corresponds to Cu atoms with + II oxidation state acknowledging that the oxidation state is an approximation and does not corresponds to the partial charge of atoms. Using this approach within the Heikes formalism, often used in the case of degenerate semiconductors^26^, the following formula of the Seebeck coefficient is obtained:

1 $${\boldsymbol{S}}=\frac{{{\boldsymbol{k}}}_{{\boldsymbol{B}}}}{{\boldsymbol{q}}}\,ln[(\frac{{{\boldsymbol{g}}}_{1}}{{{\boldsymbol{g}}}_{2}})\frac{[{\boldsymbol{C}}{{\boldsymbol{u}}}^{+}]}{[{\boldsymbol{C}}{{\boldsymbol{u}}}^{2+}]}]$$

where g_1_ = 1 and g_2_=4 are electron degeneracies and of Cu^+^ and Cu^2+^ while [Cu^+^]/[Cu^2+^] is the ratio of Cu having +I and +II oxidation states, acknowledging that the oxidation state doesn’t correspond to the spatial charge of atoms. This formula allows the calculation of Cu^2+^ fraction sites and furthermore p, the carriers concentration. Finally, the mobility μ can be estimated using **σ = peµ**. The results are presented in Table 1. It should be mentioned here that for conductive samples (σ > 1 S cm^−1^) the band degenerate^29^ and the small polaron models lead to similar values^14^. Moreover for lower conductive samples a good agreement is observed using both small polaron and non-degenerate models^30^. The logarithmic dependence of the carriers’ concentration and drift mobilities is depicted in Figs. 2(e and f) for both annealing processes. The decrease of the carriers’ concentration is efficiently tailored by controlling the annealing temperature, as in this case a smoother variation is observed. However, if low values are aimed at, the fast annealing may be privileged. An interesting observation is related to the drift mobility. During both thermal treatments a minimum is observed: after 900 s of heating at 800 °C or after 30 s of heating at 900 °C. This behaviour might be related to the atomic rearrangement during the annealing process. In these conditions, the number of grain boundaries may increase to a maximum and furthermore scattering the carrier flow.

**Figure 3:**
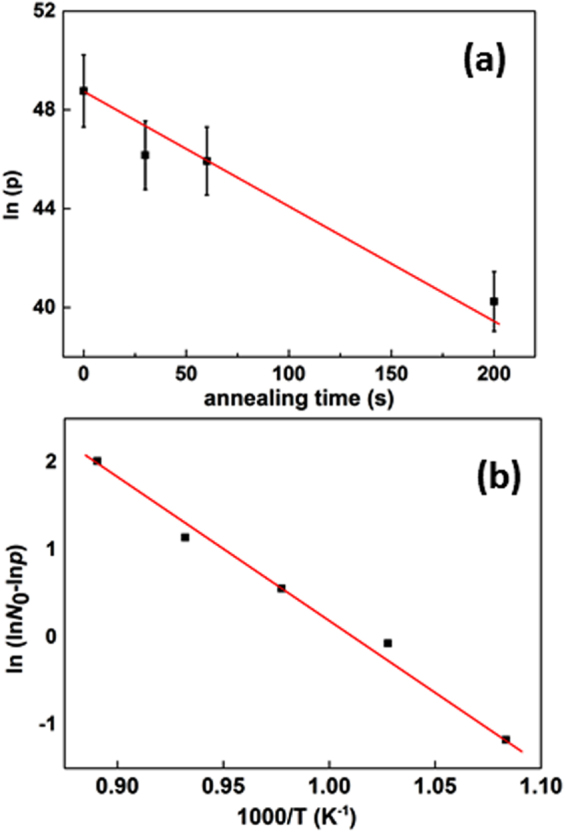
**(a)** Natural logarithm of the carrier concentration as a function of annealing time for Cu-Cr-O films annealed at a fixed temperature T = 1173 K; **(b)** Arrhenius plot of the difference of the natural logarithm of carrier concentration and initial doping, $\mathrm{ln}\,{{\rm{N}}}_{0}-\,\mathrm{ln}\,{\rm{p}}$, as a function of the annealing temperature for CuCrO_2_ annealed during a fixed time t = 900 s. The size of error bar is comparable with the symbol ‘size. Red lines: linear fits to the data.

The results obtained after the two thermal processes described above are also used to investigate the energetics of doping defects. Assuming a first order kinetics law, the holes concentration *p* can be expressed as a function of the annealing time *t* in a general way as:

2 $${\rm{p}}({\rm{t}})\approx {{\rm{N}}}_{{\rm{a}}}({\rm{t}})={{\rm{N}}}_{{\rm{i}}}{{\rm{e}}}^{-{\rm{kt}}}+{{\rm{N}}}_{{\rm{res}}}\equiv {{\rm{N}}}_{0}{{\rm{e}}}^{-{\rm{kt}}}+{{\rm{N}}}_{{\rm{res}}}{\mathrm{with}N}_{{\rm{o}}}\gg {{\rm{N}}}_{{\rm{res}}}$$

where N_a_ is the acceptor dopant concentration, N_res_ is the residual acceptor concentration after an infinite annealing, N_i_ = N_0_ + N_res_ is the initial carrier concentration (as-deposited) and k is the rate constant. The assumption of a first order kinetic is validated by the observed linear dependence when plotting ln(p) vs. t for the data taken at 900 °C (Fig. 3(a)). Since the data point at 1000 s is already very close to the residual holes concentration p_res_, only data up to 200 s of annealing are fitted. Consequently a rate constant *k* = 0.040 ± 0.005 s^−1^ is obtained. Assuming that the reaction is thermally activated, the reaction constant k can be expressed as a function of the annealing temperature T using an activation energy E_a_:

3 $${\rm{k}}({\rm{T}})={{\rm{Ae}}}^{-\frac{{{\rm{E}}}_{{\rm{a}}}}{{{\rm{k}}}_{{\rm{B}}}{\rm{T}}}}$$

where A is a constant and k_B_ is the Boltzmann constant. Relating equations (2) and (3), we can express the carrier concentration as a function of the annealing temperature *T* and annealing time t:

4 $${\rm{p}}({\rm{T}},{\rm{t}})={{\rm{N}}}_{0}\,\exp (-{\rm{At}}\,\exp (-\frac{{{\rm{E}}}_{{\rm{a}}}}{{{\rm{k}}}_{{\rm{B}}}{\rm{T}}}))+{{\rm{N}}}_{{\rm{res}}}$$

The residual dopant concentration can be neglected and the relationship can be thus expressed:

5 $$\mathrm{ln}(\mathrm{ln}\,{{\rm{N}}}_{0}-\,\mathrm{ln}\,{\rm{p}}({\rm{T}},{\rm{t}}))=\,\mathrm{ln}({\rm{At}})-\frac{{{\rm{E}}}_{{\rm{a}}}}{{{\rm{k}}}_{{\rm{B}}}{\rm{T}}}$$

Taking the as-deposited carrier concentration as the initial dopant concentration N_i_ = N_0_ = 1.68×10^21^ cm^−3^, we can plot $\mathrm{ln}(\mathrm{ln}\,{{\rm{N}}}_{0}-\,\mathrm{ln}\,{\rm{p}}({\rm{T}},{\rm{t}}))$ at fixed annealing time (t = 900) s as a function of 1/*T* (Fig. 3(b)).

The data fit well with the activation energy model, and an activation energy of E_a_ = 1.35 ± 0.07 eV is obtained. In the case of ZnO^31^ the activation energy for the annihilation of Frenkel pairs (annihilation of vacancies with nearby interstitials) is about 1 eV while the energy barriers for the migration of zinc and oxygen vacancies are 1.4 eV and 2.4 eV, respectively. Based on this quantitative comparison and on our previous microscopic analysis of defects showing the presence of Cu vacancy chains in as-deposited material, we suggest that the changes in electrical conductivities in our system are triggered by Ostwald ripening mechanisms based on the dissolution of shorter chains of vacancies into single Cu vacancies migrating to grain boundaries or longer chained vacancy defects. In our case, the activation energy would preferably correspond to the energy of migration of single Cu vacancies through the crystal. A detailed analysis of the structural evolution of defects goes beyond the present study but it is noteworthy that our measured activation is in the same range as the migration energy of cations vacancy in oxide semiconductors^32,33^.

**Figure 4:**
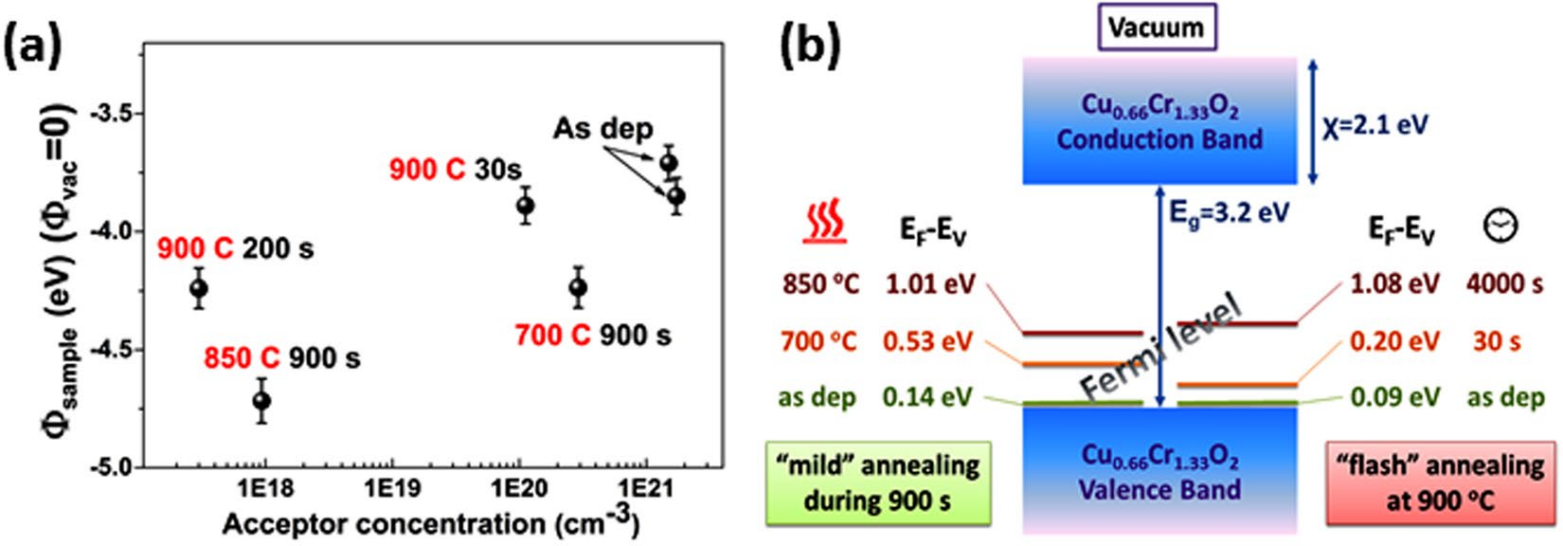
(**a**) work-function difference as a function of the carrier concentration); **(b)** calculated Fermi level for delafossite film annealing at different temperatures and for various time intervals.

KPFM measurements were performed on six samples: both reference samples plus two samples from the first set (900 s, 700 °C and 850 °C) and two from the last set (900 °C, 30 s and 4000 s). The measurements were performed alternatively between the HOPG reference and one of the samples. The values are always compared to the latest reference value to avoid possible fluctuations of the tip work function (e.g. due to contaminations). In order to compensate the vacuum levels misalignment KPFM insert a voltage V_DC_ = (Φ_tip_ − Φ_sample_)/e where Φ_tip(Pt-Ir)_ = 5.5 eV. The samples have different doping levels and different Fermi levels (E_F_) are expected. An increase of the work function ΔΦ is measured according to:

6 $${E}_{F}-{E}_{V}=({\rm{{\rm X}}}+{E}_{g})-\Delta \Phi $$

where χ is the electronic affinity (2.1 eV for copper delafossites^34^), Eg is the band gap 3,2 eV^2,12,14^. This value is insignificantly affected by annealing^14^ and thus holds for both cases of annealed and as-deposited (transmittance spectra and Tauc plots for as deposited and annealed samples are presented in Figure S5). The results are depicted in Fig. 4(a), where the work-function referenced to the vacuum (knowing that Φ_HOPG_ = 4.6 eV) is shown as a function of the carriers’ concentration. For as deposited samples, the Fermi level is situated at 90 meV above the valence band maximum, an ordinary value for degenerate semiconductors. Upon thermal treatment the Fermi level can be tailored upwards to 1.08 eV above the valence band maximum; this corresponds to the samples annealed for 4000 s at 900 °C (Fig. 4(b)). The Fermi level is thus gradually tailored within this range of energy (90 meV to 1.08 eV) when the thermal treatment is tuned.

## Conclusions

We proved that the controlled thermal treatment is an efficient approach for controlling electronic properties of Cu_0.66_Cr_1.33_O_2_, a highly conductive p-type transparent oxide. The holes concentration, the electrical mobility and the electronic work function are key features for transparent solid-state devices and for a suitable integration in any active devices (such as p-n junction, transistors or optoelectronic devices) these properties must be tailored in order to tune the required properties of such devices. Therefore we showed that the carrier concentration was smoothly varied from 10^21^ to 10^17^ cm^−3^, the electrical conductivity swaps down 5 orders of magnitude while the recorded changes in drift mobility covered three orders of magnitude. Moreover, we accurately control the Fermi level which is of critical importance in controlling the carrier injection and extraction in electronic devices. Depending on the aimed final values we proposed a time driven or a temperature driven thermal treatment. From the value of 90 meV for as-deposited samples, the Fermi level can be tailored upwards up to 1.08 eV above the valence band maximum; this is achieved via the annealing of as-deposited sample for 4000 s at 900 °C. The energetics of singular Cu-vacancies chain defects responsible of the high conductivity in this peculiar material is also studied. An activation energy model is used to extract the averaged activation energy of the annihilation of Cu-vacancies chains. We found E_a_ = 1.35 eV which is very similar to the energy of the migration of Zn-vacancies in n-type ZnO-based TCOs.

## Methods

Thin films with a thickness around 200 nm were deposited on Al_2_O_3_ c-cut substrates using a Dynamic Liquid Injection - Metal Organic Chemical Vapour Deposition system (DLI-MOCVD, MC200 from Annealsys) whilst bis 2,2,6,6-tetramethyl-3,5-heptanedionate compounds were used as precursors for copper and chromium. The deposition parameters are: temperature substrate = 450 °C; oxygen flow = 2000 standard cubic centimetres (sccm); nitrogen flow = 850 sccm; total process pressure = 12 mbar. A detailed description of the deposition approach fabrication can be found elsewhere^35^. The annealing processes were performed in a Rapid Thermal Annealing reactor (Annealsys) under Argon, under vacuum or at high oxygen partial pressure (in same conditions as during the deposition) and the changes in conductivity were not significantly depending on the annealing atmosphere. For the present work the annealing was performed under N_2_ + O_2_ mixture for the fast annealing and under vacuum for the annealing at various temperatures. Electrical properties were measured using four probes in linear configuration while for the Seebeck effect measurement a homemade system was used with a copper wire as reference. A detailed description of this system is presented on supplementary information section **(**Figure S5**)**. For X-Ray Photoemission Spectroscopy (XPS) analysis a Kratos Axis Ultra DLD system using a monochromated (Al Kα: hν = 1486.7 eV) X-ray source was used. Kelvin Probe Force Microscopy (KPFM) measurements have been performed on a Bruker Innova using the amplitude modulation to determine the contact potential difference between the Pt-Ir coated tip and the sample surface. The analysis is done in one pass mode, the first resonance amplitude is used to track the topography while the KPFM signal is acquired at a second frequency (23 kHz). Freshly cleaved Highly-Oriented Pyrolytic Graphite (HOPG) is used as reference. Measurements are performed under dry N_2_ atmosphere in order to avoid water condensation on the surface.

## Electronic supplementary material

Supplementary information
