# Supplementary material for: Tuning the electrical properties of the p-type transparent conducting oxide Cu1−xCr1+xO2 by controlled annealing
Source: Sci Rep. 2018 May 8;8:7216. doi: 10.1038/s41598-018-25659-3 (PMC5940695; doi:10.1038/s41598-018-25659-3)
Supplement: Supplementary file 1 — Supplementary information [file 41598_2018_25659_MOESM1_ESM.docx]

**Supplementary Material**

**Tuning the electrical properties of the p-type transparent conducting oxide Cu_1-x_Cr_1+x_O by controlled annealing**

*P. Lunca-Popa, J. Afonso, P. Grysan, J. Crêpellière, R. Leturcq and D. Lenoble*

Materials Research and Technology Department (MRT), Luxembourg Institute of Science and Technology (LIST), 41 rue de Brill, L-4422 Belvaux, Luxembourg

Corresponding author: [petru.luncapopa@list.lu](mailto:petru.luncapopa@list.lu)

**Figure S1.** GIXRD spectra for films annealed at 900 ^º^C for different periods of time.

Due to the small size of the crystalline grains, grazing incidence XRD was used in order to collect information from a bigger volume of thin-films, and to detect most of the crystalline planes.


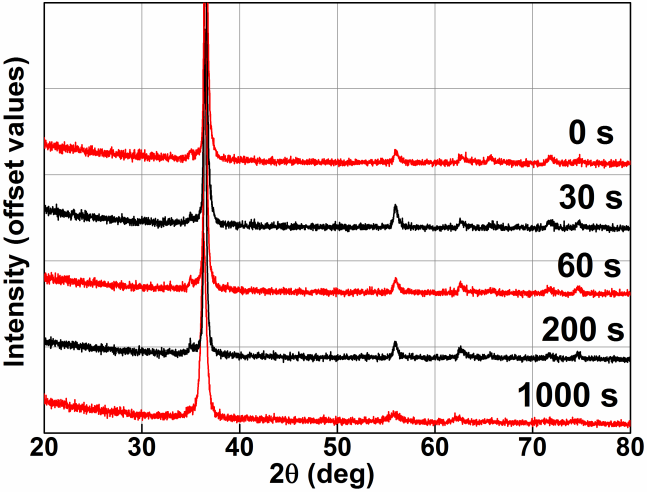


**Figure S2.** GIXRD spectra for as-deposited and for the films annealed at 900 ^º^C for 4000s. Inset: refined view for the most intense diffraction peaks. A small displacement to bigger angles is observed


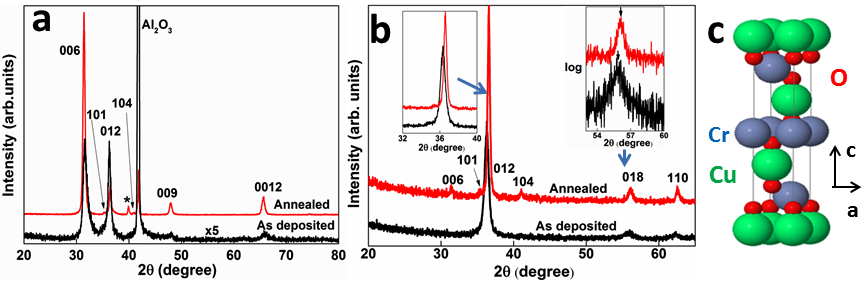


**Figure** **S3.** AFM scans for films annealed for 0, 200 and 1000 s respectively. The horizontal scale bar is 1 micron

*
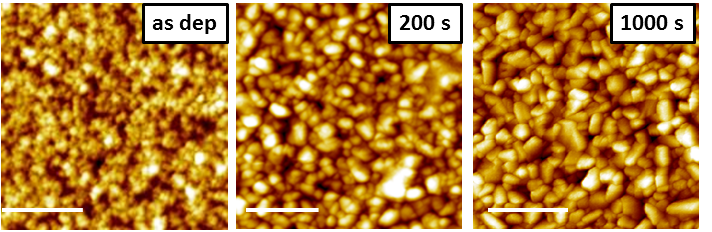
*

**Figure** **S4.** Transmittance spectra and Tauc plots for samples annealed at 900 ^o^C for different time intervals


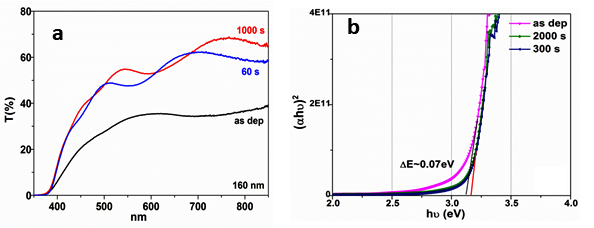


**Figure S5** Experiment set-up for measuring Seebeck coefficient

**
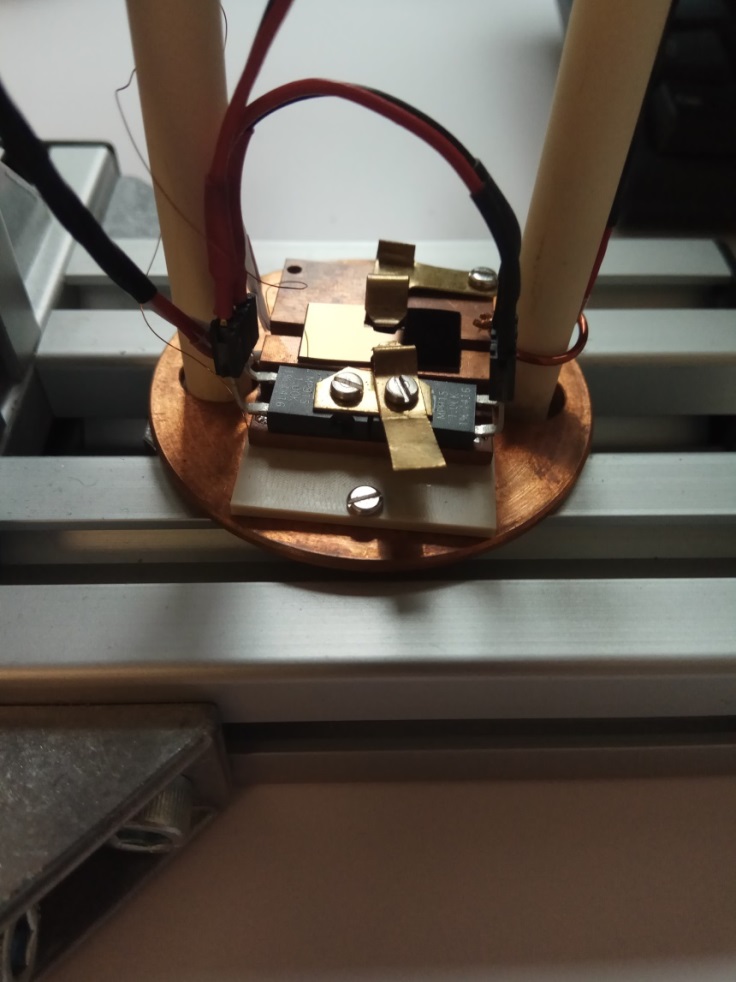
**

**Description of Seebeck coefficient experimental set-up**

The set-up is made of two copper pieces attached to each other through a plate in order to avoid thermal and electrical conduction between each piece. The larger copper piece is thermalized to the base temperature (room temperature during the experiments here), while the second smaller copper piece is heated through a resistive heater. The temperature of each piece is measured by two calibrated Pt100 resistive thermometers embedded in the copper pieces and thermally connected to them by Apiezon® grease. The thermo-voltage is measured by a high impedance source-measure unit Keithley 2634B, directly between each copper piece using a thermalized copper wire, which serves as reference material. The sample is mechanically pressed on both copper pieces, with the deposited film being directly in contact to each copper piece. We have further checked a reference by measuring the thermo-voltage of a thin copper wire placed at the sample position.
